# Combined classification system based on ACR/EULAR and ultrasonographic scores for improving the diagnosis of Sjögren's syndrome

**DOI:** 10.1371/journal.pone.0195113

**Published:** 2018-04-03

**Authors:** Yukinori Takagi, Hideki Nakamura, Misa Sumi, Toshimasa Shimizu, Yasuko Hirai, Yoshiro Horai, Ayuko Takatani, Atsushi Kawakami, Sato Eida, Miho Sasaki, Takashi Nakamura

**Affiliations:** 1 Department of Radiology and Cancer Biology, Nagasaki University Graduate School of Biomedical Sciences, Nagasaki, Japan; 2 Department of Immunology and Rheumatology, Nagasaki University Graduate School of Biomedical Sciences, Nagasaki, Japan; 3 Clinical Research Center, National Hospital Organization (NHO) Nagasaki Medical Center, Nagasaki, Japan; Technische Universitat Munchen, GERMANY

## Abstract

We retrospectively evaluated the effectiveness of combined use of salivary gland ultrasonography (US) and the 2016 American College of Rheumatology/European League Against Rheumatic Disease (ACR/EULAR) classification criteria for improving the diagnostic efficiency in patients with Sjögren’s syndrome (SS). A US-based salivary gland disease grading system was developed using a cohort comprising 213 SS or non-SS patients who fulfilled the minimum requirements for classifying SS based on the American-European Consensus Group (AECG) and ACR criteria. Using 62 SS or non-SS patients from the 213 patients and who had also undergone all the 5 examinations needed for the ACR/EULAR classification, we compared the diagnostic accuracy of various combinations of the ACR/EULAR and US classifications for diagnosing SS, using the clinical diagnosis of SS by rheumatologists as the gold standard. The ACR/EULAR criteria discriminated clinical SS patients with 77% and 79% accuracy for those with primary or secondary SS and for those with primary SS, respectively. However, the integrated score system of the ACR/EULAR and US classifications yielded 92% and 93% accuracy for these 2 SS patient groups, respectively, provided that US score of 3 was assigned to patients with US grade ≥2, and then patients with integrated threshold score of ≥5 were diagnosed as SS. Cross-validation also indicated improved accuracy of the integrated ACR/EULAR and US score system (91.9 and 93.0% for primary/secondary and primary SS patients, respectively) over that by the ACR/EULAR criteria alone. (74.2 and 86.0%, respectively). The integrated ACR/EULAR and US scoring system can improve the diagnosis of patients with clinical SS.

## Introduction

Several classification systems for Sjögren’s syndrome (SS) have been proposed, validated, and updated by the American, European, and Japanese societies. In particular, the most recently published American College of Rheumatology (ACR)/European League Against Rheumatic Diseases (EULAR) classification uses a simplified classification hierarchy and the scoring system for diagnosing patients with primary SS [1].

However, SS has basically no ‘gold standard’, and, in the clinics the final diagnosis is usually made by experienced rheumatologists after analyzing clinical data, including demographic properties, symptoms, and the results from serological, histological and imaging examinations of patients with possible SS [2]. Therefore, there may be substantial discrepancies between a diagnosis based on established classification criteria used and the clinical diagnosis of SS by a rheumatologist. Accordingly, several attempts have been made to improve the diagnostic accuracy of the SS classification criteria and to match the diagnostic results to those of the ‘gold standard’ by the rheumatologists [3–5].

One possible strategy for improving the diagnostic accuracy of an established SS classification system is to incorporate an imaging classification item in the system, such as ultrasonography (US), which was adopted for effectively discriminating the glands of SS patients from those of non-SS patients with clinical symptoms of xerostomia/xerophthalmia [6–8]. An additional benefit of incorporating an imaging item in an SS classification system (particularly the ACR/EULAR criteria) is that the imaging item can provide a scale for evaluating the severity of gland disease [7, 9]. This approach, however, has not yet achieved good results. Considering the high accessibility, the light financial burden both on patients and physicians, no need for radiation exposure, US should be a strong candidate for this purpose. However, inconsistency in interpreting the US images of the salivary glands has been a practical problem; the same image is often classified in different ways by different readers, and is differently classified by the same reader at different times.

In the present study, we retrospectively evaluated the effectiveness of combined use of the salivary gland US and the ACR/EULAR classification criteria for improving the diagnosis of patients with SS using the clinical diagnosis of rheumatologists as a reference standard. Also in this attempt, we carefully developed a US-based grading system for the severity of salivary gland disease in SS patients, based on logistic regression models that fitted the probability of SS, thereby reasonably decreasing the inter- and intraobserver errors in reading US images. We report here the improved diagnostic accuracy for SS provided by an integrated score system based on salivary gland US and the ACR/EULAR classification criteria.

## Materials and methods

### Patients

We used two study cohorts: (1) 213 patients who fulfilled the minimum requirements for diagnosing SS or non-SS according to both the AECG classification and the ACR classification criteria; and (2) 40 patients with SS and 22 patients without SS patients comprising the subset of cohort 1 (n = 213) who had undergone all the 5 clinical and laboratory tests needed for the ACR/EULAR classification-based SS diagnosis. The large cohort, which was used for developing a US-based salivary gland disease grading system, comprised 133 patients with SS (124 women and 9 men; average age, 57 ± 15 years) and 80 patients without SS (63 women and 17 men; average age, 60 ± 13 years) when diagnosed based on the AECG criteria; these patients were diagnosed as having SS (n = 128, 120 women and 8 men; average age, 58 ± 14 years) or as not having SS (n = 85, 67 women and 18 men; average age, 59 ± 14 years) when diagnosed based on the ACR criteria. The 133 AECG-diagnosed SS patients consisted of 85 primary SS and 48 secondary SS patients; and the 128 ACR-diagnosed SS patients consisted of 78 primary SS and 50 secondary SS patients.

The smaller sub-cohort was used for assessing the diagnostic accuracy of single or combined criteria of the ACR/EULAR and salivary gland US classifications for diagnosing SS as stated below, and was composed of 42 patients with primary or secondary SS (39 women and 3 men; average age, 56 ± 16 years) and 20 patients without SS (16 women and 4 men; average age, 59 ± 12 years) patients according to the ACR/EULAR criteria. Of these, 28 were diagnosed as having primary SS (25 women and 3 men; average age, 55 ± 16 years) and 15 were diagnosed as not having SS (13 women and 2 men; average age, 60 ± 14 years).

The study protocol was approved by the ethics committee of Nagasaki University Hospital, and informed consent was waived for the retrospective nature of the study.

### Characterization of US imaging features of the salivary glands

Gray-scale US of the parotid and submandibular glands was performed at 10 MHz using a Logiq 700 or a Logiq 9 unit with a wide bandwidth (6–14 MHz) (GE Healthcare). US images obtained through the transverse planes of the parotid and submandibular glands were analyzed by 3 radiologists who had 10- to 12-years experiences in the field of US diagnosis of the salivary glands of patients with SS and who were blind to the clinical information of the patients. The list of US findings suspicious of SS and the occurrence rates of the findings in the present study cohorts (n = 213) are shown in S1 **Table**. The final decision was made by consensus of the 3 radiologists.

### Logistic regression analysis and SS probability assessment of US findings

Univariate and multivariable analyses were performed to investigate the relationship between US findings and SS diagnosis, which was made based on the AECG or ACR classification criteria [10, 11]. In the multivariable analysis, significant US findings identified in the univariate analysis were incorporated into a regression model. Odds ratios and 95% confidence intervals (CI) were calculated. Based on the results from the logistic regression analysis, we determined important US findings for diagnosing SS (**Table 1**). We used one (hypoechoic area, for the ACR-based SS classification) or two (hypoechoic area and hyperechoic band, for the AECG-based SS classification) US imaging items for evaluating the parotid glands; and three (hypoechoic area, hyperechoic band, and irregular border, for the ACR and AECG-based SS classification) items for evaluating the submandibular glands. A score (0 for negative and 1 for positive US findings) was assigned for each gland image.

**Table 1:** Diagnostic items for US scoring system.

US findings	Patients diagnosed based on the classification criteria of			
	AECG		ACR	
	parotid gland	submandibular gland	parotid gland	submandibular gland
hypoechoic area	+ (1.65)	+ (1.91)	+ (2.20)	+ (1.98)
hyperechoic band	+ (0.52)	+ (0.69)	-	+ (0.65)
irregular border	-	+ (0.89)	-	+ (1.36)

+/-, used (+) or not used (-) for the diagnosis

One (hypoechoic area for ACR-based SS classification) or two items (hypoechoic area and hyperechoic band for AECG-based SS classification) were used for the parotid glands, and three items (hypoechoic area, hyperechoic band, and irregular border, for AECG and ACR-based SS classification) were used for the submandibular glands for the US scoring as determined by the multivariable logistic regression analysis (**S2 Table**).

Figures in parentheses indicate the standardized regression coefficients (β) that are obtained for each US finding from the multivariable logistic regression analysis.

The US score was multiplied (weighted) by the standardized regression coefficient (β), which was obtained for each significant US finding from the multivariable logistic regression analysis. Then, an equation that fitted the probability of SS diagnosis was obtained separately for the AECG- or ACR-based SS diagnosis. Probability of SS per gland was calculated by the following equation:

$$Probability=1/[1+\exp\left(-P_{L})\right]$$ (1)

where P_L_ (logit) = log[p/(1-p)]. The P_L_ value was calculated using the corresponding regression model for each gland. A score sum from the 4 salivary glands (2 parotid and 2 submandibular glands) was calculated in each patient (**Table 2**). Patients were categorized into either of the 5 ascending US grades (0–4) with increasing probabilities of SS, separately for patients who were diagnosed based on the AECG- or ACR-criteria. The risk of SS was determined according to the following equation:

Risk(%)=[no.ofSSpatients]×100/[totalno.ofpatientsineachUSgrade] (2)

**Table 2:** US score system and probability and risk of SS in SS or non-SS patients who are compatible with AECG or ACR criteria.

AECG-based classification					
US grade^a^	0	1	2	3	4
US score sum^b^	0	1–2	3–5	6–8	9–10
no. of patients (SS/nonSS)	7/21	17/31	21/17	39/7	49/4
Probability (mean ± s.d.)^c^	0.322	0.412 ± 0.048	0.570 ± 0.092	0.785 ± 0.069	0.876 ± 0.020
Probability (range)^c^	—	0.358–0.541	0.438–0.768	0.652–0.848	0.805–0.884
Risk of SS (%)^d^	25.0	35.4	55.3	84.8	92.5
					
ACR-based classification					
US grade^a^	0	1	2	3	4
US score sum^b^	0	1–2	3–5	6–7	8
no. of patients (SS/non-SS)	3/25	20/36	21/16	30/6	54/2
Probability (mean ± s.d.)^c^	0.271*	0.380 ± 0.055*	0.593 ± 0.109*	0.777 ± 0.073*	0.879*
Probability (range)^c^	—	0.298–0.495	0.406–0.782	0.645–0.863	—
Risk of SS (%)^d^	10.7	35.7	56.8	83.3	96.4

SS, Sjögren’s syndrome; AECG, American-European Consensus Group classification; ACR, American College of Rheumatology classification.

a, Patients were categorized into either of the 5 ascending US grades (0–4) with increasing probabilities of SS, separately for patients who were diagnosed based on the AECG or ACR classification criteria.

b, US score sum is a total score of the 4 salivary glands (2 parotid and 2 submandibular glands) from a single SS or non-SS patient and is calculated separately for different SS criteria (AECG or ACR); the full score differs between different SS criteria (AECG and ACR).

c, *Probability per gland* = 1/[1 + exp(−*P_L_*)], where *P_L_*(logit) = log[1/(1 − P)]. *P_L_* = −0.33 + 1.65 × [hypoechoic area] + 0.52 × [hyperechoic band] for the parotid glands; and *P_L_* = −1.23 + 1.91 × [hypoechoic area] + 0.69 × [hyperechoic band] + 0.89 × [irregular border] for the submandibular glands in AECG-based US scoring system. *P_L_* = −0.51 + 2.20 × [hypoechoic area] for the parotid glands; and *P_L_* = −1.61 + 1.98 × [hypoechoic area] + 0.65 × [hyperechoic band] + 1.36 × [irregular border] for the submandibular glands in ACR-based US scoring system. *Probability per patient* = [*probability sum from the* 4 *salivary glands* (2 × *PGs and* 2 × *SMGs*)]/4

d, *Risk* (%) = [*no. of SS patients*]/[*total no. of patients in each US grade*]

*, Significant differences between different US grades (p <0.001, Steel-Dwass test).

For all regression calculations, a p-value <0.05 was considered significant.

### ACR/EULAR criteria scores

The ACR/EULAR classification scores were calculated for the 62 patients derived from the 213 SS or non-SS patients according to the published report [1], assigning score 3 for positive results of the labial gland biopsy and of the serological test for anti-SSA/Ro antibodies, and score 1 for positive results of the ocular staining test, Schirmer’s test, and the salivary flow rate test. Although the original ACR/EULAR classification was established for the primary SS patients, we diverted this classification system to suit the secondary SS patients as well.

### Integrated score system for the combination of ACR/EULAR and US

**Fig 1:**
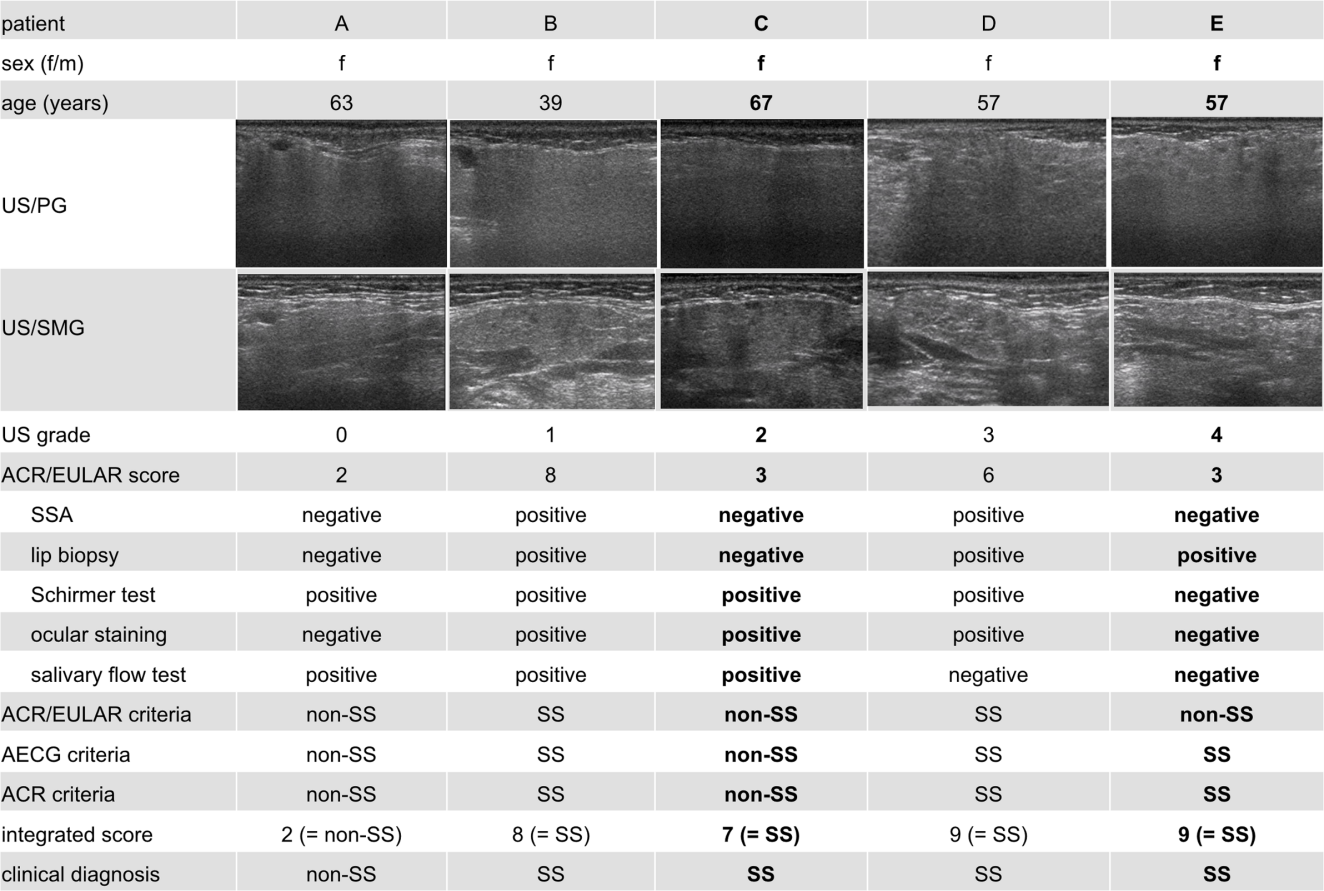
Representative US images/scores, ACR/EULAR scores, and integrated ACR/EULAR plus US scores vs. clinical SS diagnosis. Five patients (**A**-**E**) were clinically diagnosed as SS or non-SS by rheumatologists (clinical diagnosis). US grades were calculated as described in the text. ACR/EULAR scores were calculated based on the 5 test (serological test for anti-SS-A antibodies, labial salivary gland biopsy, ocular staining test, and salivary flow test) as previously described [1], except for the salivary flow test, which was performed by Saxon test. Patients were diagnosed as SS or non-SS based on the ACR/EULAR criteria (threshold score ≥4) or on the integrated ACR/EULAR plus US scores (threshold score ≥5, using assigned US score of 3) as described in the text. Patients were also classified into SS or non-SS based on the AECG or ACR classification criteria as previously described [10, 11]. Two patients (**C** and **E**) whose results are shown in bold were those with different classification results between the ACR/EULAR criteria and the clinical diagnosis by rheumatologists, but with the same results between the integrated ACR/EULAR plus US criteria and the clinical diagnosis.

To construct the integrated score system for combined criteria of the ACR/EULAR and the salivary gland US classifications, variable salivary gland US scores of 1 to 3 were assigned to patients who had salivary gland US grades that were considered positive for SS; US score 0 was assigned to patients with US grades considered negative for SS, and US score of 1 to 4 was assigned to patients with US grades that were considered positive for SS (**Tables 3** and **4**). The final classification of SS was made using varying thresholds of integrated scores of the ACR/EULAR (0 to 9) plus the salivary gland US (0 to 3) classifications. The final classifications based on the integrated ACR/EULAR and US score system were compared with the clinical diagnosis of SS by rheumatologists (**Fig 1**).

**Table 3:** Diagnostic ability of combined classification system of 2016 ACR/EULAR and AECG criteria-based US scores for SS in 40 SS and 22 non-SS patients^a^.

system components		US threshold	US score assigned	integrated threshold	diagnostic ability^b^	
ACR/EULAR	US	grade 0–4	grade 0–4	score 0–12	AECG	ACR
ACR/EULAR (score threshold ≥4)						
(+)	(−)	—	—	—	85/64/77	
US grade (grade threshold, ≥1 to ≥4)						
(−)	(+)	—	≥1	—	90/23/66	90/23/66
(−)	(+)	—	≥2	—	80/68/76	75/73/74
(−)	(+)	—	≥3	—	58/100/73	53/100/69
(−)	(+)	—	≥4	—	38/100/60	40/100/61
simple combination: ACR/EULAR (score threshold ≥4) + US (grade threshold ≥1 to ≥4)						
(+)	(+)	≥1	—	—	80/73/77	80/73/77
(+)	(+)	≥2	—	—	70/91/77	65/91/74
(+)	(+)	≥3	—	—	55/100/71	50/100/68
(+)	(+)	≥4	—	—	33/100/56	38/100/60
integrated combination: ACR/EULAR [score 0–9] + US [score 0–3] (score sum threshold, ≥4 to ≥7)^c^						
(+)	(+)	≥1	1	≥4	90/55/77	90/55/77
(+)	(+)	≥2	1	≥4	90/64/81	90/64/81
(+)	(+)	≥3	1	≥4	88/64/79	88/64/79
(+)	(+)	≥4	1	≥4	88/64/79	88/64/79
(+)	(+)	≥1	1	≥5	85/68/79	85/68/79
(+)	(+)	≥2	1	≥5	85/86/85	83/86/84
(+)	(+)	≥3	1	≥5	78/91/82	78/91/82
(+)	(+)	≥4	1	≥5	78/91/82	78/91/82
(+)	(+)	≥1	2	≥4	95/45/77	95/45/77
(+)	(+)	≥2	2	≥4	95/64/84	95/64/84
(+)	(+)	≥3	2	≥4	90/64/81	90/64/81
(+)	(+)	≥4	2	≥4	90/64/81	88/64/79
(+)	(+)	≥1	2	≥5	90/59/79	90/59/79
(+)	(+)	≥2	2	≥5	**90/86/89**	**88/86/87**
(+)	(+)	≥3	2	≥5	80/91/84	80/91/84
(+)	(+)	≥4	2	≥5	80/91/84	80/91/84
(+)	(+)	≥1	2	≥6	80/73/77	80/73/77
(+)	(+)	≥2	2	≥6	80/91/84	78/91/82
(+)	(+)	≥3	2	≥6	73/100/82	73/100/82
(+)	(+)	≥4	2	≥6	73/100/82	73/100/82
(+)	(+)	≥1	3	≥4	95/27/71	95/27/71
(+)	(+)	≥2	3	≥4	95/50/79	95/50/79
(+)	(+)	≥3	3	≥4	90/64/81	90/64/81
(+)	(+)	≥4	3	≥4	90/64/81	88/64/79
(+)	(+)	≥1	3	≥5	95/50/79	95/50/79
(+)	(+)	≥2	3	≥5	**95/86/92**	**93/86/90**
(+)	(+)	≥3	3	≥5	83/91/85	83/91/85
(+)	(+)	≥4	3	≥5	83/91/85	80/91/84
(+)	(+)	≥1	3	≥6	85/64/77	85/64/77
(+)	(+)	≥2	3	≥6	85/91/87	83/91/85
(+)	(+)	≥3	3	≥6	75/100/84	75/100/84
(+)	(+)	≥4	3	≥6	75/100/84	75/100/84
(+)	(+)	≥1	3	≥7	80/73/77	80/73/77
(+)	(+)	≥2	3	≥7	80/91/84	78/91/82
(+)	(+)	≥3	3	≥7	73/100/82	73/100/82
(+)	(+)	≥4	3	≥7	68/100/79	65/100/77

a, Clinical diagnosis of SS or non-SS was made by consensus of 6 rheumatologists.

b, Diagnostic abilities are shown separately for AECG or ACR criteria-based US grading and expressed in sensitivity/specificity/accuracy (sens/spec/acc).

c, Patients were categorized into SS-positive or -negative based on the US score sum from the bilateral parotid and submandibular glands using varying US grade (0–4) thresholds. Variable US scores (1–3 for patient with positive US glands images) were assigned to patients positive for SS and US score 0 to those negative for SS. Then, the final diagnosis of SS was made using varying thresholds of integrated ACR/EULAR plus US scores (4–12 for positive results) and was compared with the clinical SS diagnosis by the rheumatologists. Two results with the best diagnostic accuracy in each of the AECG- or ACR-based US score systems are shown in bold.

**Table 4:** Diagnostic ability of combined classification system of 2016 ACR/EULAR and AECG criteria-based US scores for primary SS in 23 SS and 20 non-SS patients^a^.

system components		US threshold	US score assigned	integrated threshold	diagnostic ability^b^	
ACR/EULAR	US	grade 0–4	grade 0–4	score 0–12	AECG	ACR
ACR/EULAR (score threshold ≥4)						
(+)	(−)	—	—	—	91/65/79	
US grade (grade threshold, ≥1 to ≥4)						
(−)	(+)	—	≥1	—	91/25/60	91/25/60
(−)	(+)	—	≥2	—	78/70/74	74/70/72
(−)	(+)	—	≥3	—	52/100/74	48/100/72
(−)	(+)	—	≥4	—	39/100/67	39/100/67
simple combination: ACR/EULAR (score threshold ≥4) + US (grade threshold ≥1 to ≥4)						
(+)	(+)	≥1	—	—	83/85/84	83/85/84
(+)	(+)	≥2	—	—	78/70/74	74/70/72
(+)	(+)	≥3	—	—	48/100/72	43/100/70
(+)	(+)	≥4	—	—	35/100/65	35/100/65
integrated combination: ACR/EULAR [score 0–9] + US [score 0–3] (score sum threshold, ≥4 to ≥7)^c^						
(+)	(+)	≥1	1	≥4	100/55/79	100/55/79
(+)	(+)	≥2	1	≥4	100/65/84	100/65/84
(+)	(+)	≥3	1	≥4	96/65/81	96/65/81
(+)	(+)	≥4	1	≥4	96/65/81	96/65/81
(+)	(+)	≥1	1	≥5	91/70/81	91/70/81
(+)	(+)	≥2	1	≥5	91/85/88	87/85/86
(+)	(+)	≥3	1	≥5	78/90/84	78/90/84
(+)	(+)	≥4	1	≥5	78/90/84	78/90/84
(+)	(+)	≥1	2	≥4	100/45/74	100/45/74
(+)	(+)	≥2	2	≥4	100/65/84	100/65/84
(+)	(+)	≥3	2	≥4	96/65/81	96/65/81
(+)	(+)	≥4	2	≥4	96/65/81	96/65/81
(+)	(+)	≥1	2	≥5	100/60/81	100/60/81
(+)	(+)	≥2	2	≥5	**100/85/93**	**96/85/91**
(+)	(+)	≥3	2	≥5	87/90/88	87/90/88
(+)	(+)	≥4	2	≥5	87/90/88	87/90/88
(+)	(+)	≥1	2	≥6	83/75/79	83/75/79
(+)	(+)	≥2	2	≥6	83/90/86	78/90/84
(+)	(+)	≥3	2	≥6	74/100/86	74/100/86
(+)	(+)	≥4	2	≥6	74/100/86	74/100/86
(+)	(+)	≥1	3	≥4	100/35/70	100/35/70
(+)	(+)	≥2	3	≥4	100/60/81	100/60/81
(+)	(+)	≥3	3	≥4	96/75/86	96/75/86
(+)	(+)	≥4	3	≥4	96/75/86	96/75/86
(+)	(+)	≥1	3	≥5	100/50/77	100/50/77
(+)	(+)	≥2	3	≥5	**100/85/93**	**96/85/91**
(+)	(+)	≥3	3	≥5	87/90/88	87/90/88
(+)	(+)	≥4	3	≥5	87/90/88	87/90/88
(+)	(+)	≥1	3	≥6	91/65/79	91/65/79
(+)	(+)	≥2	3	≥6	91/90/91	87/90/88
(+)	(+)	≥3	3	≥6	78/100/88	78/100/88
(+)	(+)	≥4	3	≥6	78/100/88	78/100/88
(+)	(+)	≥1	3	≥7	83/75/79	83/75/79
(+)	(+)	≥2	3	≥7	83/90/86	78/90/84
(+)	(+)	≥3	3	≥7	74/100/86	74/100/86
(+)	(+)	≥4	3	≥7	74/100/86	74/100/86

a, Clinical diagnosis of SS or non-SS was made by consensus of 6 rheumatologists.

b, Diagnostic abilities are shown separately for AECG or ACR criteria-based US grading and expressed in sensitivity/specificity/accuracy (sens/spec/acc).

c, Patients were categorized into SS-positive or -negative based on the US score sum from the bilateral parotid and submandibular glands using varying US grade (0–4) thresholds. Variable US scores (1–3 for patient with positive US glands images) were assigned to patients positive for SS and US score 0 to those negative for SS. Then, the final diagnosis of SS was made using varying thresholds of integrated ACR/EULAR plus US scores (4–12 for positive results) and was compared with the clinical SS diagnosis by the rheumatologists. Two results with the best diagnostic accuracy in each of the AECG- or ACR-based US score systems are shown in bold.

### Clinical diagnosis of SS by rheumatologists

Clinical diagnosis of SS was performed by 6 Japanese College of Rheumatology (JCR)-board certified rheumatologists on the cohort of 62 ACR/EULAR compatible patients, based on the clinical and laboratory data including demographic properties, clinical symptoms, results from clinical and laboratory tests of blood and saliva samples, imaging findings from sialography and scintigraphy, and histological findings from labial gland biopsy. The rheumatologists were blinded to the results from US examinations. The final clinical diagnosis of SS made by consensus of the 6 JCR-board certified rheumatologists was regarded as the ‘gold standard’.

### Cross-validation

The diagnostic accuracy of the single or combined criteria for clinically diagnosed SS patients was assessed using cross-validation. In the 62 primary/secondary SS or non-SS patient group, 7-fold cross-validation was performed by partitioning the patients into 7 subsets of equal (or nearly equal) size, and then using a different fold, in turn, for testing, and the remaining 6 folds for training. In the primary SS group that was comprised of 28 primary SS and 15 non-SS patients, 6-fold cross validation was performed in a similar fashion.

### Statistical analysis

Logistic regression analysis was performed by using the Statistical Package for Social Sciences (SPSS) version 18 software. Differences in fitted probabilities between patients with different US grades were assessed using the Steel-Dwass test by using the JMP Pro version 13 software. Cross-validation was performed by using the MatLab software version R2017a. P values <0.05 were considered statistically significant in all the tests.

## Results

### Establishment of US-based salivary gland grading system for SS

Based on the multivariable regression analysis results (**S2 Table**), we categorized the salivary glands (bilateral parotid and submandibular glands) into ascending 5 grades (0–4) with increasing probabilities of the SS diagnosis that fitted the multivariable regression models, separately for the AECG- or ACR-based SS classification (**Table 2**, **Fig 1**). Typically, the 213 patients were categorized into 5 grades with risks (and probabilities) of 10.7% (0.271), 35.7% (0.380), 56.8% (0.593), 83.3% (0.777), and 96.4% (0.879) based on the ACR criteria for SS. Similar results with higher sensitivity for SS prediction were obtained for the AECG-based classification (**Table 2**). The fitted probabilities were significantly different between any two US grades, whether categorized based on either the AECG- or ACR-based scheme (p <0.001, Steel-Dwass test).

### Diagnostic accuracy of single or combined classification criteria for clinical SS diagnosis

We then compared the diagnostic accuracy of single or combined criteria of the ACR/EULAR and/or salivary gland US classifications within the sub-cohort of 62 primary/secondary SS or non-SS patients (**Table 3**). The ACR/EULAR classification diagnosed clinical SS patients with 85% sensitivity, 64% specificity, and 77% accuracy. However, the integrated score system of the ACR/EULAR and US classifications best provided 95% sensitivity, 86% specificity, and 92% accuracy for the AECG-based US grading; and 93% sensitivity, 86% specificity, and 90% accuracy for the ACR-based US grading, provided that a US score of 3 was assigned to a patient with US grade of ≥2, and then a patient with an integrated ACR/EULAR plus US score of ≥5 was diagnosed as having SS. In contrast, the US grade criteria alone or simple combination of the ACR/EULAR and US classification criteria (i.e., positive for both the criteria systems), did not improve the diagnostic accuracy over that by the ACR/EULAR classification.

The ACR/EULAR classification was originally established for the diagnosis of primary SS patients. Therefore, we next assessed the diagnostic accuracy of the single or combined criteria within the primary SS or non-SS patient group (**Table 4**). Again, the integrated score system of the ACR/EULAR and salivary gland US classifications achieved the best results, yielding 100% sensitivity, 85% specificity, and 93% accuracy for the AECG-based US grading; and 96% sensitivity, 85% specificity, and 91% accuracy for the ACR-based US grading, provided that US score of 3 was assigned to a patient with US grade of ≥2, and then a patient with a resulting integrated ACR/EULAR plus US score of ≥5 was diagnosed as having SS. However, in this case the identical results were also obtained when US score of 2 was assigned to a patient with US grade of ≥2, and a patient with an integrated ACR/EULAR plus US scores of ≥5 was diagnosed as SS (**Table 4**).

### Cross-validation

The study cohort used for evaluating the diagnostic accuracy was relatively small. Therefore, we performed cross-validation analysis to confirm the diagnostic accuracy. Seven-fold cross-validation performed on the primary/secondary SS patient sub-cohort indicated that the diagnostic accuracy using the ACR/EULAR classification criteria for clinical SS was 74.2% (**Table 5**). However, the accuracy using the integrated score system of the ACR/EULAR and US classifications was 91.9% for the AECG-based US classification and 90.3% for the ACR-based US classification provided that a US score of 3 was assigned to a patient with US grade of ≥2. In contrast, the other criteria tested did not improve the diagnostic accuracy over that of the ACR/EULAR classification criteria.

**Table 5:** Cross-validation of single or combined criteria for SS.

classification	US score assigned	cross-validation (%)^a^	
	score 0–3	US grading based on	
		AECG	ACR
primary/secondary SS or non-SS (n = 62: 42 SS and 20 non-SS patients)			
ACR/EULAR	—	74.2	
US	—	72.6	71.0
simple combination	—	77.4	74.2
integrated combination	2	88.7	87.1
	3	**91.9**	**90.3**
primary SS or non-SS (n = 43: 28 SS and 15 non-SS patients)			
ACR/EULAR	—	86.0	
US	—	72.1	65.1
simple combination	—	79.1	76.7
integrated combination	2	**93.0**	**90.7**
	3	**93.0**	88.4

a, Diagnostic accuracy of the single or combined criteria for clinically diagnosed SS patients was assessed using cross-validation. Seven- and six-fold cross-validation analyses were performed for the 62 primary and secondary SS (primary/secondary SS) or non-SS group and 43 primary SS or non-SS groups, respectively. The best results with the AECG- or ACR-based US grading in each of the patient groups (primary/secondary SS or non-SS, and primary SS or non-SS) are indicated in bold.

Similar results were obtained in the primary SS or non-SS patient group, where the best results were achieved by the integrated score system of the ACR/EULAR and US classifications when a US score of 2 or 3 was assigned to a patent with US grade of ≥2 for the AECG-based US classification, and when US score of 2 was assigned to a patient with US grade of ≥2 for the ACR-based US classification (**Table 5**).

## Discussion

In the present study, we showed that an integrated score system based on the ACR/EULAR and salivary gland US classifications for SS can greatly improve on the diagnostic accuracy of the original ACR/EULAR criteria, approximating to the clinical diagnosis of SS by sophisticated rheumatologists. However, US alone, or a simple combination of the ACR/EULAR- and US grading-based classifications did not surpass the diagnostic accuracy of the ACR/EULAR criteria.

Several attempts have been made to improve on the diagnostic accuracy of established SS criteria. Among those, Cornec et al. tested the possibility of improving diagnostic ability by adding a US grading system that scaled the salivary gland disease severity to the AECG classification criteria, using clinical diagnosis by experts as the reference standard [3]. They introduced a regression model to verify the diagnostic items such as salivary flow rate, Schirmer’s test, salivary gland biopsy, and serological test (anti-SSA/SSB antibodies), as well as the US grading, and then compared diagnostic abilities between the single AECG and combined criteria. They found that the addition of US criteria increased the sensitivity from 78% to 86% at the expense of specificity, which was decreased from 99% to 95%. Similar results were reported by the same study group for SS or non-SS patients who were diagnosed based on the ACR criteria [4]. In the present study, however, we showed that an integrated score system based on the ACR/EULAR plus US classification, but not a simple combination of these classification criteria, increased the specificity without any loss of sensitivity.

Most recently, Mossel et al. have shown that a combination of parotid and submandibular gland US grading and a serological test for anti-SS antibodies can effectively predict the AECG, ACR, or ACT/EULAR classification results of patients [5]. The authors reported that the absolute agreement of US results was at most moderate compared with an AECG (82%), ACR (86%), or ACR/EULAR (80%) based classification, as well as with labial gland biopsy (79%) results. These results imply that a combination of US and these diagnostic items could improve diagnostic accuracy. Our present data support this hypothesis concerning the effectiveness of combining the ACR/EULAR and US classification criteria.

The integrated score system proposed in the present study is based on the ACR/EULAR and US classifications and uses an assigned US score of 3 and an integrated score threshold of ≥5 (**Table 6**). The ACR/EULAR criteria includes five classification items, of which two (serological and lip biopsy) had a score of 3 each, and the remaining three had a score of 1 each. The threshold score for SS is 5. Therefore, you should have at least one positive item having a score of 3 in order to reach the threshold. To keep this aspect of the original ACR/EULAR criteria for the proposed score system, the threshold should be higher than 5, and/or the assigned score for the US classification should be lower. For example, a patient with dry mouth/eyes might be diagnosed as having SS without face validity for an autoimmune basis of the disease. However, the other combinations of score and score threshold tested did not surpass the ability to duplicate the reference standard by the rheumatologists (**Tables 3** and **4**).

**Table 6:** Integrated score system based on the ACR/EULAR and US classifications.

classification item	score assigned
anti-SS-A/Ro antibody	3
labial gland biopsy	3
Schirmer’s test	1
ocular staining	1
salivary flow test^a^	1
salivary gland US^b^	3
integrated score threshold for SS	≥5

The integrated score system is available for diagnosing

primary and secondary SS.

a, Saxon’s test was performed in the present study.

b, Score 3 is assigned to a patient with US grade ≥2 as described in the text. The score system for the other classification items was described previously [1].

Different US scoring systems have been used in different ways to differentiate between SS and non-SS patients, with success [3, 4, 12–15]. However, the consensus is that total US scores for parotid and submandibular glands are more effective compared to scores from either of the gland types alone [16]. To establish an appropriate scoring system, logistic regression analysis was used for weighting the diagnostic items used in the regression models. We also used a logistic regression analysis to this end. In addition, the score system used in the present study was intended to facilitate a simplified scoring system that would be convenient for the physicians who manage the care of SS patients. Accordingly, we have taken advantages of using the strategy to establish an imaging reporting and data system for US features [17], thereby allowing the categorization of US images of the parotid and submandibular glands from a possible SS patient into either of the 5 grades with ascending scores from 0 to 4, based on the summed scores obtained from both types of the bilateral major salivary glands.

The most difficult problem, however, may be that the interobserver agreement was not high (indicated by low κ-values) for some US finding charactereistics of the salivary glands of patients with SS. In this regard, Jousse-Joulin et al. reported that interobserver agreements were fair to substantial (κ-values = 0.20 to 0.66) [18]. To overcome the low reliability of specific US imaging findings, the final decision in the present study was made by consensus of the 3 expert radiologists. The introduction of a machine learning (deep learning) tool could potentially alleviate the problem of low interobserver agreement, if a machine could learn more from the US images of the salivary glands of SS or non-SS patients than physicians can, and could use the knowledge extracted from the images to make better decisions than the physicians do [19].

The clinical diagnosis of SS by rheumatologists has been used as a gold standard [3–5, 15]. The clinical diagnosis of SS by physicians is usually based on broad and different criteria, including demographic data, clinical findings, laboratory and serological tests of blood and saliva samples, sialography, scintigraphy, and histopathology of labial gland specimens. Some of the items are reportedly highly correlated with US findings, but others are not [13, 20]. More importantly, the clinical diagnosis depends on the physician’s skill and experience. In fact, a given patient who is suspected of having SS is often classified differently by different physicians, and sometimes by the same physician at different times. In the present study, the final diagnosis of clinical SS was made by consensus of the sophisticated (JCR certified) rheumatologists. It would be more convenient, however, if we could use a machine learning tool in this aspect too of SS diagnosis.

A major limitation of this study is the small cohort size of SS and non-SS patients who underwent all the clinical or laboratory tests involved in the ACR/EULAR classification system. The original patient list from the institutional data base included 1956 SS or non-SS patients who received US examination. However, the inclusion criteria requiring patients who were compatible with the AECG and ACR classifications, and requiring patients having the results from all 5 ACR/EULAR criteria items, drastically reduced the available cohort size. Accordingly, we performed cross-validation to verify the reliability of the results obtained from the small cohort. However, a ramification of the cohort into primary and secondary SS groups, and the relatively small numbers of non-SS patients, may greatly bias the results. As mentioned above, the noticeable level of interobserver errors that occurred in analyzing the salivary gland US images may be another problem in a clinical setting.

The diagnostic validity may be an issue for the case where one gland is scored 3 with 3 positive US findings suggestive of SS, and thus the US grade 2 is assigned to the patient (**Table 2**). In that case, a score of 3 for the proposed integrated score system (**Table 6**) will be given to the patient based on only one pathological gland, irrespective of the disease states of the other 3 salivary glands (parotid and submandibular glands) of the patient. Therefore, the presence of only one pathological gland with a score of 3 in the integrated system (where the score threshold for SS is ≥5) would greatly affects the final diagnosis of SS. This might introduce a bias in diagnosing SS. However, the gland disease severities match between the same gland type on the left and right sides of the same patient. Therefore, the gland on the opposite side of the pathological gland with a score of 3 is similarly damaged and scored. Accordingly, the US score sum of a patient having a pathological gland with score 3 must be higher than 3. This means that the US grade of the patient must be higher than 2 (**Table 2**). In that case, the pathological gland with a score of 3 must be the submandibular gland, because the maximum number of positive US criteria item is 3 for the submandibular gland, but only 1 or 2 for the parotid gland (**Table 1**). Actually, in our study cohort the submandibular glands had the same score of 3 in 65 of the 213 patients; 60 and 62 patients of these were diagnosed as having SS based on the AECG and ACR criteria, respectively. However, the left and right submandibular glands were differently scored in 19 patients with one gland scored 3 and the other 2; 17 and 14 patients of these were diagnosed as having SS based on the AECG and ACR criteria, respectively. Only one SS patient had the submandibular gland scored 3 for one gland and 0 for the other; this patient was diagnosed as having SS based both on the AECG and ACR criteria.

Recently, US has been used for predicting treatment efficacy and clinical activity [16, 21, 22]. Furthermore, US elastography, including acoustic radiation force impulse imaging, has been shown to be useful for diagnosing SS and predicting the treatment outcomes pf SS patients [23, 24]. It can, threrfore, be expected that an integrated score system based on the ACR/EULAR and US classifications could predict the activity of SS and treatment efficacy in response to cevimeline/pilocarpine or rituximab/infliximab [25]. However, the integrated score system proposed in the present study did not include any diagnostic item relating to blood flow kinetics in the salivary glands, which could be useful for assessing the treatment efficacy [21, 26]. In this regard, D’Agostino et al. recently proposed possible paths of algorithms for the use of US in diagnosing and follow-up of patients with rheumatoid arthritis (RA) [27]. US could also potentially be used for these purposes in managing SS patients, employing similar types of algorithms. To address these questions, it would be valuable to carry out a prospective study using a large cohort of possible SS patients who are compatible with the ACR/EULAR classification-based SS diagnosis.

In conclusion, we have presented an integrated scoring system, based on the ACR/EULAR classification and a proposed US grading system, for the clinical diagnosis of SS. We found that the integrated scoring system greatly improved the ability to replicate the clinical diagnosis of SS by experienced rheumatologists. Therefore, the system could be used as an updated diagnostic tool for SS.

## Supporting information

S1 Table Occurrence rates of US findings characteristic of SS salivary glands. a, Numbers indicate bilateral parotid and/or submandibular glands from 213 SS or non-SS patients.
US, ultrasonography; SS, Sjögren’s syndrome; AECG, American-European Consensus Group classification; ACR, American College of Rheumatology classification; PG, parotid gland; SMG, submandibular gland
b, Chi-square test. P-values <0.05 were considered statistically significant (bold values).
(DOCX)

S2 Table Logistic regression analysis of US findings independently predictable of SS. US, ultrasonography; SS, Sjögren’s syndrome; 95% CI, 95% confidence interval; PG, parotid gland; SMG, submandibular gland. AECG, American European Consensus Group classification; ACR, American Rheumatology College classification. Logistic regression analysis was performed separately for AECG- or ACR-based SS diagnosis. P-values <0.05 were considered statistically significant (bold values).
(DOCX)
