# Supplementary material for: Combined classification system based on ACR/EULAR and ultrasonographic scores for improving the diagnosis of Sjögren's syndrome
Source: PLoS One. 2018 Apr 3;13(4):e0195113. doi: 10.1371/journal.pone.0195113 (PMC5882118; doi:10.1371/journal.pone.0195113)
Supplement: S1 Table — a, Numbers indicate bilateral parotid and/or submandibular glands from 213 SS or non-SS patients. US, ultrasonography; SS, Sjögren’s syndrome; AECG, American-European Consensus Group classification; ACR, American College of Rheumatology classification; PG, parotid gland; SMG, submandibular gland b, Chi-square test. P-values <0.05 were considered statistically significant (bold values). (DOCX) [file pone.0195113.s001.docx]

**S1 Table**. **Occurrence rates of US findings characteristic of SS salivary glands**

|  | SS | | non-SS | | p-value^b^ |
| --- | --- | --- | --- | --- | --- |
|  | AECG^a^  n = 266 | ACR^a^  n = 256 | AECG^a^  n = 160 | ACR^a^  n = 170 | SS vs. non-SS  (AECG/ACR) |
|  | no. of PG or SMG (%) | | | |  |
| PG |  |  |  |  |  |
| increased/decreased echogenicity | 24 (9) | 23 (9) | 16 (10) | 17 (10) | 0.7233/0.7112 |
| heterogeneous parenchyma | 157 (59) | 160 (63) | 40 (25) | 37 (22) | **<0.001/<0.001** |
| hypoechoic area | 171 (64) | 173 (68) | 34 (21) | 32 (19) | **<0.001/<0.001** |
| large (≥ 2 mm) hypoechoic area | 151 (57) | 156 (61) | 30 (19) | 25 (15) | **<0.001/<0.001** |
| hyperechoic band | 118 (44) | 118 (46) | 26 (16) | 26 (15) | **<0.001/<0.001** |
| cystic change | 0 (0) | 0 (0) | 0 (0) | 0 (0) | — |
| dilated duct | 1 (0.004) | 1 (0.004) | 0 (0) | 0 (0) | — |
| calcification | 0 (0) | 0 (0) | 0 (0) | 0 (0) | — |
| SMG |  |  |  |  |  |
| increased/decreased echogenicity | 28 (11) | 28 (11) | 13 (8) | 13 (8) | 0.4223/0.2613 |
| heterogeneous parenchyma | 197 (74) | 195 (76) | 44 (28) | 46 (27) | **<0.001/<0.001** |
| hypoechoic area | 205 (77) | 202 (79) | 36 (23) | 39 (23) | **<0.001/<0.001** |
| large (≥ 2 mm) hypoechoic area | 185 (70) | 185 (72) | 31 (19) | 31 (18) | **<0.001/<0.001** |
| hyperechoic band | 189 (71) | 185 (72) | 48 (30) | 52 (31) | **<0.001/<0.001** |
| irregular gland border | 181 (68) | 184 (72) | 53 (33) | 50 (29) | **<0.001/<0.001** |
| cystic change | 0 (0) | 0 (0) | 0 (0) | 0 (0) | — |
| dilated duct | 0 (0) | 0 (0) | 0 (0) | 0 (0) | — |
| calcification | 0 (0) | 0 (0) | 0 (0) | 0 (0) | — |

a, Numbers indicate bilateral parotid and/or submandibular glands from 213 SS or non-SS patients.

US, ultrasonography; SS, Sjögren’s syndrome; AECG, American-European Consensus Group classification; ACR, American College of Rheumatology classification; PG, parotid gland; SMG, submandibular gland

b, Chi-square test. P-values <0.05 were considered statistically significant (bold values).
