# Supplementary material for: Combined classification system based on ACR/EULAR and ultrasonographic scores for improving the diagnosis of Sjögren's syndrome
Source: PLoS One. 2018 Apr 3;13(4):e0195113. doi: 10.1371/journal.pone.0195113 (PMC5882118; doi:10.1371/journal.pone.0195113)
Supplement: S2 Table — US, ultrasonography; SS, Sjögren’s syndrome; 95% CI, 95% confidence interval; PG, parotid gland; SMG, submandibular gland. AECG, American European Consensus Group classification; ACR, American Rheumatology College classification. Logistic regression analysis was performed separately for AECG- or ACR-based SS diagnosis. P-values <0.05 were considered statistically significant (bold values). (DOCX) [file pone.0195113.s002.docx]

**S2 Table. Logistic regression analysis of US findings independently predictable of SS**

| US criteria items | no. of glands | | | odds ratio (95% CI) | | | |
| --- | --- | --- | --- | --- | --- | --- | --- |
|  |  | SS | | univeriate | | multivariable | |
|  |  | (+) | (−) |  | p-value |  | p-value |
| **AECG-based** |  |  |  |  |  |  |  |
| PG |  |  |  |  |  |  |  |
| irregular echogenicity | (+) | 24 | 16 | 0.89 (0.46-1.74) | 0.739 | — | — |
|  | (−) | 242 | 144 |  |  |  |  |
| hypoechogenic area | (+) | 157 | 40 | 6.67 (4.24-10.51) | **<0.001** | 9.33 (3.77-23.13) | **<0.001** |
|  | (−) | 109 | 120 |  |  |  |  |
| hyperechogenic band | (+) | 118 | 26 | 4.11 (2.53-6.67) | **<0.001** | 2.05 (1.10-3.82) | **0.0238** |
|  | (−) | 148 | 134 |  |  |  |  |
| heterogeneous parenchyma | (+) | 157 | 40 | 4.32 (2.80-6.66) | **<0.001** | 0.46 (0.17-1.19) | 0.0952 |
|  | (−) | 109 | 120 |  |  |  |  |
| SMG |  |  |  |  |  |  |  |
| irregular echogenicity | (+) | 28 | 13 | 1.33 (0.67-2.65) | 0.411 | — | — |
|  | (−) | 238 | 147 |  |  |  |  |
| hypoechogenic area | (+) | 205 | 36 | 11.58 (7.25-18.49) | **<0.001** | 9.28 (3.99-21.59) | **<0.001** |
|  | (−) | 61 | 124 |  |  |  |  |
| hyperechogenic band | (+) | 189 | 48 | 5.73 (3.73-8.80) | **<0.001** | 2.21 (1.26-3.87) | **0.0064** |
|  | (−) | 77 | 112 |  |  |  |  |
| heterogeneous parenchyma | (+) | 197 | 44 | 7.53 (4.84-11.71) | **<0.001** | 0.64 (0.26-1.58) | 0.3262 |
|  | (−) | 69 | 116 |  |  |  |  |
| irregular border | (+) | 181 | 53 | 4.30 (2.83-6.53) | **<0.001** | 2.47 (1.52-4.01) | **<0.001** |
|  | (−) | 85 | 107 |  |  |  |  |
|  |  |  |  |  |  |  |  |
| **ACR-based** |  |  |  |  |  |  |  |
| PG |  |  |  |  |  |  |  |
| irregular echogenicity | (+) | 23 | 17 | 0.89 (0.46-1.72) | 0.726 | — | — |
|  | (−) | 233 | 153 |  |  |  |  |
| hypoechogenic area | (+) | 173 | 32 | 8.99 (5.65-14.31) | **<0.001** | 9.14 (3.86-21.64) | **<0.001** |
|  | (−) | 83 | 138 |  |  |  |  |
| hyperechogenic band | (+) | 118 | 26 | 4.74 (2.92-7.69) | **<0.001** | 1.87 (0.99-3.51) | 0.0533 |
|  | (−) | 138 | 144 |  |  |  |  |
| heterogeneous parenchyma | (+) | 160 | 37 | 5.99 (3.84-9.34) | **<0.001** | 0.69 (0.28-1.75) | 0.4302 |
|  | (−) | 96 | 133 |  |  |  |  |
|  |  |  |  |  |  |  |  |
| SMG |  |  |  |  |  |  |  |
| irregular echogenicity | (+) | 28 | 13 | 1.48 (0.75-2.95) | 0.253 | — | — |
|  | (−) | 228 | 157 |  |  |  |  |
| hypoechogenic area | (+) | 202 | 39 | 12.57 (7.88-20.04) | **<0.001** | 8.35 (3.63-19.19) | **<0.001** |
|  | (−) | 54 | 131 |  |  |  |  |
| hyperechogenic band | (+) | 185 | 52 | 5.91 (3.86-9.05) | **<0.001** | 1.99 (1.12-3.54) | **0.0200** |
|  | (−) | 71 | 118 |  |  |  |  |
| heterogeneous parenchyma | (+) | 195 | 46 | 8.62 (5.53-13.43) | **<0.001** | 0.82 (0.34-2.00) | 0.6659 |
|  | (−) | 61 | 124 |  |  |  |  |
| irregular border | (+) | 184 | 50 | 6.13 (4.00-9.41) | **<0.001** | 3.90 (2.38-6.39) | **<0.001** |
|  | (−) | 72 | 120 |  |  |  |  |

US, ultrasonography; SS, Sjögren’s syndrome; 95% CI, 95% confidence interval; PG, parotid gland; SMG, submandibular gland. AECG, American European Consensus Group classification; ACR, American Rheumatology College classification.

Logistic regression analysis was performed separately for AECG- or ACR-based SS diagnosis.

P-values <0.05 were considered statistically significant (bold values).
